# Progression of Structural Lung Disease in Different *Aspergillus fumigatus* Disease Phenotypes in Children with CF

**DOI:** 10.3390/jof11100689

**Published:** 2025-09-23

**Authors:** Federico Mollica, Eleni-Rosalina Andrinopoulou, Beyza Y. Ikiz, Punitkumar Makani, Harm A. W. M. Tiddens, Daan Caudri

**Affiliations:** 1 Department of Pediatric Pulmonology and Allergology, Erasmus MC—Sophia Children’s Hospital, Dr. Molewaterplein 40, 3015 GD Rotterdam, The Netherlands; f.mollica@erasmusmc.nl (F.M.); b.ikiz@erasmusmc.nl (B.Y.I.); p.makani@erasmusmc.nl (P.M.); h.tiddens@erasmusmc.nl (H.A.W.M.T.); 2 Department of Pediatric Pulmonology and Allergology, Erasmus MC—Sophia Children’s Hospital, Dr. Molewaterplein 40, 3015 GD Rotterdam, The Netherlands; 3 Department of Biostatistics, Erasmus MC—Sophia Children’s Hospital, Dr. Molewaterplein 40, 3015 GD Rotterdam, The Netherlands; e.andrinopoulou@erasmusmc.nl; 4 Department of Epidemiology, Erasmus MC—Sophia Children’s Hospital,Dr. Molewaterplein 40, 3015 GD Rotterdam, The Netherlands; 5 Thirona B.V., Toernooiveld 300, 6525 EC Nijmegen, The Netherlands

**Keywords:** ABPA, *Aspergillus fumigatus*, chest computed tomography, cystic fibrosis, spirometry-controlled CT

## Abstract

*Aspergillus* *fumigatus* (Asp) is frequently cultured from airways of children with Cystic Fibrosis (CwCF), but the impact on structural lung disease (SLD) remains unknown. In this retrospective study of 125 CwCF with a positive Asp airway culture (Asp+) at Sophia Children’s Hospital between 1988 and 2021, four Asp disease phenotypes were defined based on serum Asp-specific IgE (IgE_Asp_) and IgG (IgG_Asp_): colonisation, sensitisation, bronchitis, and allergic bronchitis. SLD was assessed on biennial chest CTs (n = 382) using the PRAGMA-CF score. Annual progression of SLD was modelled for the Asp disease phenotypes, adjusting for *Pseudomonas aeruginosa* and Allergic Bronchopulmonary Aspergillosis (ABPA). Annual SLD progression was high in all phenotypes but was higher in Asp sensitisation and bronchitis compared to colonisation. The proportion of air trapping was high in the full study population (mean 57%), but no differences were found in annual progression between the different Asp disease phenotypes. CwCF with Asp allergic bronchitis had a 10-fold higher risk to develop ABPA during the study follow-up than those with Asp colonisation. The four Asp disease phenotypes, colonisation, sensitisation, bronchitis, and allergic bronchitis, that were defined based on IgE_Asp_ and IgG_Asp_ show different rates of progression of SLD and different risks of ABPA development.

## 1. Introduction

Cystic Fibrosis (CF) lung disease is characterised by chronic airway inflammation and infection that starts in infancy, leading to progressive structural abnormalities of airways and lung parenchyma [1,2]. Infections are predominantly bacterial, but up to two-thirds of Children with Cystic Fibrosis (CwCF) will at some point in their lifetime also grow *Aspergillus fumigatus* (Asp) from their sputum or bronchoalveolar lavage fluid [3,4,5]. This is likely an underestimation as Asp cultures are not 100% sensitive, implying the true prevalence of *Aspergillus fumigatus*-positive airway culture (Asp+) is even higher [4,6,7]. Culture positivity without clinical signs or symptoms can be defined as Asp colonisation, and this is generally not considered an indication to start anti-fungal treatment. Multiple studies have found carriage of Asp in CwCF to be associated with worse outcomes, including lower lung function and reduced quality of life [8,9]. *Aspergillus* cultured from bronchoalveolar lavage samples associated with progressive structural lung disease (SLD) in CwCF has effect sizes comparable to those of *Pseudomonas aeruginosa* (PsA) infections [5,10]. There remains ongoing debate whether these associations are causal, or simply reflect more severe underlying CF lung disease, which, e.g., due to more aggressive antibiotic treatment, could facilitate fungal growth [11]. If the association is causal, Asp treatment should obviously be considered, but the threshold to start such treatment in colonised CwCF is very high. Treatment with anti-fungal drugs would be needed for prolonged periods and these drugs can have substantial side effects [12,13]. There are no well-conducted anti-fungal trials available in CF and success rates of eradication are low/unknown [14]. Finally, there are relatively high-risk drug–drug interactions between anti-fungal and with other CF medications, in particular the cytochrome P450 (CYP450)-mediated interaction with most novel CFTR modulator therapies.

It is very difficult to prove a causal link between Asp colonisation and worse clinical outcomes using only observational data. Importantly, even in the absence of clinical symptoms, Asp+ is known to induce different immunological responses, such as raised Asp-specific IgE (IgE_Asp_) and Asp-specific IgG (IgG_Asp_). Such systemic responses could be indicative of clinically relevant infections, and they can be used to define different Asp disease phenotypes [15,16,17]. An allergic response with IgE_Asp_ raised > 0.35 kUA/L could be considered Asp sensitisation, while an anti-infective immune response with IgG_Asp_ > 27 mgA/L could suggest Asp bronchitis [18]. Any differences in disease progression between Asp disease phenotypes based on the immunologic response by the host would not likely be caused by traditional confounding factors, such as PsA or antibiotic use. We propose that differential SLD progression between the Asp disease phenotypes would be indicative of a causal role for Asp and the incited host immune response in the development of SLD.

In this study we make use of the retrospective CF cohort at the Erasmus MC Sophia Children’s Hospital to identify CwCF with Asp+ airway cultures and investigate their progression of SLD based on the Asp disease phenotype. To assess SLD we will make use of the biennial chest computed tomography (CT) scans that are performed as part of routine clinical follow-up and quantify abnormalities with the well-validated manual Perth Rotterdam Annotation Grid Morphometric Analysis (PRAGMA-CF) scoring system [19]. We hypothesise that the different Asp disease phenotypes will be associated with different trajectories of SLD development, which would support a causal role for Asp in worse SLD outcomes. Furthermore, it would offer support for a simple blood-test-based classification of Asp lung disease that could help to identify and select CwCF that could benefit most from anti-fungal treatments in the future.

## 2. Material and Methods

### 2.1. Study Design and Population

This is a retrospective observational study conducted at the Erasmus MC Sophia Children’s Hospital in Rotterdam, the Netherlands, and approved by the Dutch Medical Ethics Review Committee on 30 August 2011 (MERC number: MEC-2011-321). The study population consisted of children with CF aged 1 to 18 years who had their first documented Asp+ between 1988 and 2021. The vast majority of scans (~95%) were acquired before the introduction of modulator therapy, and all the CTs were acquired before the highly effective triple therapy. To ensure that only incident cases were included, eligibility further required at least three prior negative Asp cultures before the first positive result. Inclusion also required the availability of at least three CTs per patient, with at least one CT performed before and one after the first Asp+.

### 2.2. Data Collection and Phenotype Classification

**Figure 1:**
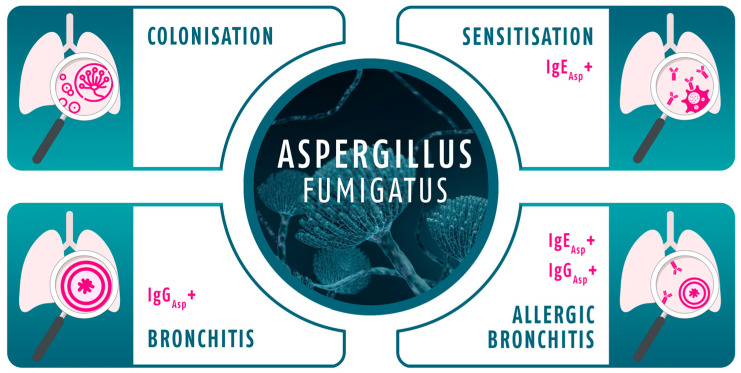
*Aspergillus fumigatus* disease phenotypes based on their serum levels of IgE_Asp_ and IgG_Asp._

Clinical and laboratory data, including microbiological cultures and serological markers, were retrieved from the hospital information system. At the time of the first Asp+, patients were categorised into four Asp disease phenotypes based on their serum levels of IgE_Asp_ and IgG_Asp_ [18]: Asp colonisation (both markers within normal range), Asp sensitisation (elevated IgE_Asp_, normal IgG_Asp_), Asp bronchitis (normal IgE_Asp_, elevated IgG_Asp_), and Asp allergic bronchitis (both markers elevated), see also Figure 1.

These phenotypes were assigned using established cut-off values [18]. The presence of PsA was defined as any positive PsA airway culture during the study period and was considered as a potential confounder in the analyses. Allergic Bronchopulmonary Aspergillosis (ABPA) was identified based on clinical diagnosis and the initiation of oral corticosteroid therapy and was treated as a cofounder rather than a separate Asp phenotype.

### 2.3. Chest CT Acquisition and Scoring

For each eligible patient, at least three and up to four CTs were included, with the requirement that at least one CT was obtained before and one after the first Asp+. All CTs were pseudonymised and manually scored at the LungAnalysis core laboratory (Erasmus MC—Sophia Children’s Hospital) using the PRAGMA-CF scoring system, a validated grid-based method for quantifying structural lung abnormalities in CF [20]. For each CT, ten equally spaced axial slices were selected, and a grid was overlapped to systematically annotate bronchiectasis, atelectasis, airway wall thickening, mucus plugging, and healthy parenchyma for the inspiratory CTs and air trapping for the expiratory CTs. The composite variable, total disease, was calculated as the sum of all airway-related abnormalities (bronchiectasis, atelectasis, airway wall thickening, and mucus plugging) [20]. All the CTs were assessed by a trained and certified PRAGMA-CF scorer (FM) in randomised order.

### 2.4. Statistical Analysis

The primary outcome of the study was the progression of SLD, assessed by serial CTs. Linear mixed-effects models were constructed to evaluate changes in PRAGMA-CF scores before and after first Asp+ for continuous outcomes and two-part hurdle mixed-effects models for semi-continuous data, with time zero defined as the date of first Asp+ for each patient. These models accounted for the correlation between the measurements taken from the same patient by including random intercepts. Both linear and non-linear (assuming natural splines) structure for the linear mixed-effects model were tested to allow for differential disease progression rates before and after Asp colonisation.

Furthermore, to assess whether disease trajectory differed by Asp phenotype, interaction terms between time and phenotype were incorporated. As sensitivity analyses, separate mixed-effects models were constructed for each Asp phenotype. Models were adjusted for the presence of PsA and ABPA, given their known association with Asp and SLD. A second sensitivity analysis excluded patients who developed ABPA to evaluate the robustness of the findings.

## 3. Results

In this retrospective study, 147 children with CF and an Asp+ airway culture between the ages of 1 and 18 years were initially screened at the time of their first ever Asp+. Of those, 125 children (males 59, 47.2%) met all inclusion criteria and contributed a total of 382 inspiratory CTs to the analysis. The median age at first Asp+ was nine years (IQR six years), and the mean interval between consecutive CTs was two years. Expiratory CTs were available for a subset of 105 children, with a total of 266 expiratory CTs.

### 3.1. Aspergillus Disease Phenotypes

Patients were classified into four Asp disease phenotypes based on IgE_Asp_ and IgG_Asp_ levels at the time of first Asp+ (see Table 1): Asp colonisation (both specific IgE_Asp_ and IgG_Asp_ normal, n = 44), Asp sensitisation (specific IgE_Asp_ elevated, IgG_Asp_ normal, n = 32), Asp bronchitis (specific IgE_Asp_ normal, IgG_Asp_ elevated, n = 10), and Asp allergic bronchitis (both specific IgE_Asp_ and IgG_Asp_ elevated, n = 39).

There was no clear association between age at first Asp+ and disease phenotype. ABPA was not classified as a separate phenotype, as children from all four specified phenotypes could develop ABPA at any point in the study period. Interestingly, children classified as Asp allergic bronchitis at the time of first Asp+ had about a threefold higher likelihood of developing ABPA during follow-up than those with sensitisation or bronchitis, and about a tenfold higher likelihood than those with colonisation (*p*-value < 0.01). The prevalence of PsA infection was high across the cohort (76.8%) and did not differ between Asp phenotypes. See Table 1 for other patients’ descriptives overall and within the Asp disease phenotypes.

### 3.2. Progression of Structural Lung Disease

**Figure A1:**
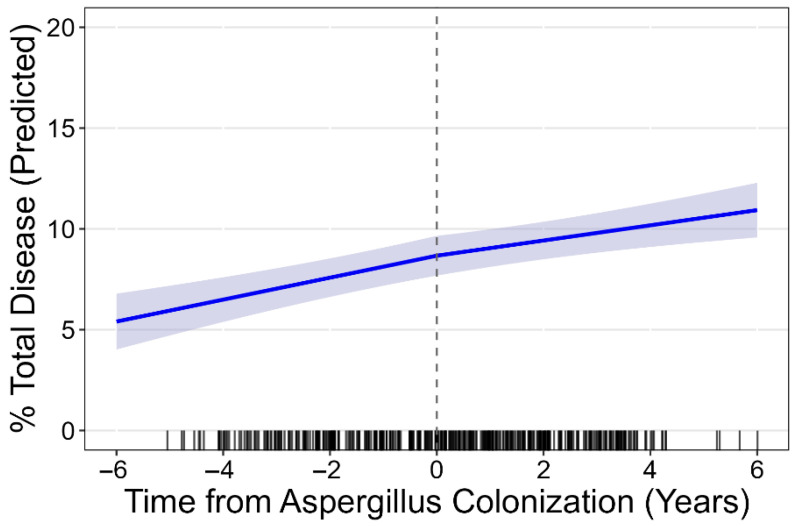
Results from spline model allowing different rates of total % disease progression before and after first Asp+ in whole study group (n = 125). Rates are not different (*p* = 0.3871).

**Figure A2:**
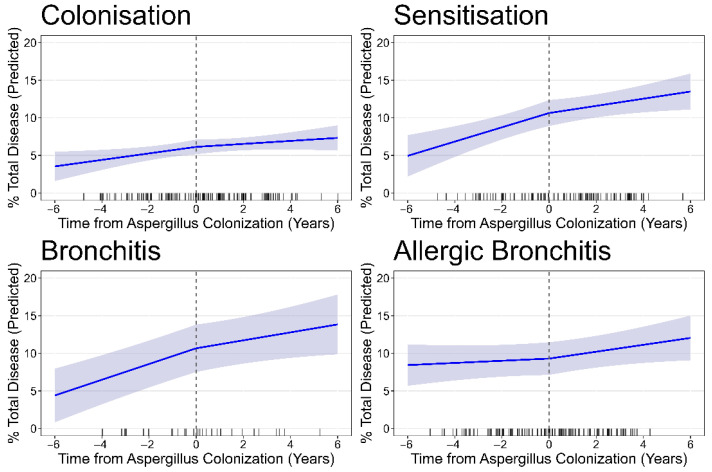
Results from spline model allowing different rates of total % disease progression before and after first Asp+ in the different Asp disease phenotypes. Colonisation (n = 44): Rates are not different (*p* = 0.4489). Sensitisation (n = 32): Rates are not different (*p* = 0.2642). Bronchitis (n = 10): Rates are not different (*p* = 0.2180). Allergic bronchitis (n = 39): Rates are not different (*p* = 0.4077).

The progression of SLD was analysed using linear mixed-effect models, which allowed for both random intercepts and the possibility of non-linear progression before and after the first Asp+. However, the data best fits a simple linear model with stable disease progression over the study period, indicating no change in the rate of SLD progression after first Asp acquisition (Figure A1). This was also found within the four disease phenotype subgroups. (Figure A2). Consequently, disease progression is modelled linearly throughout the study period in all subsequent models. Table 2 shows the prevalence of the different SLD outcomes at first moment of Asp+, as well as the unadjusted average progression per year in the whole cohort and in the different Asp disease phenotype subgroups.

**Figure 2:**
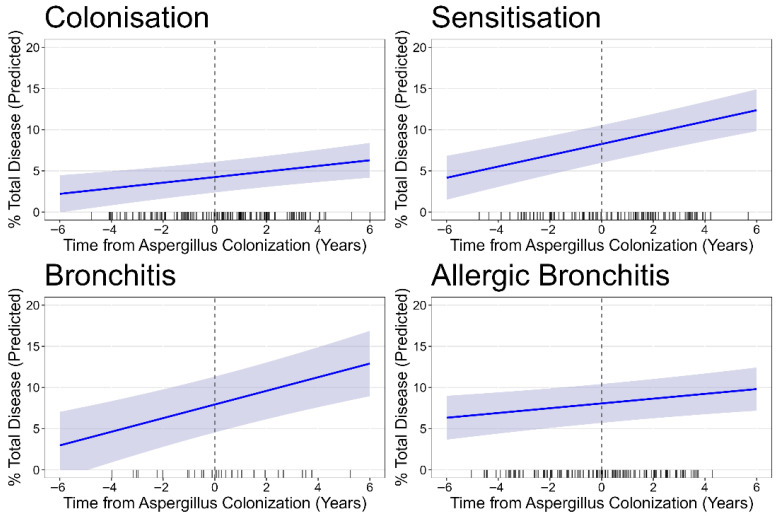
Longitudinal progression of total % disease stratified by Asp disease phenotype. The figure displays the modelled trajectory of total % disease over time, where timepoint 0 = the moment of first Asp+. Each line represents the predicted mean total % disease, with shaded areas indicating the 95% confidence intervals.

At the time of first Asp+, children with Asp sensitisation, Asp bronchitis, and Asp allergic bronchitis all exhibited higher levels of total SLD (total % disease) than those with Asp colonisation. Also, the unadjusted rates of progression per year were higher in the Asp sensitisation and bronchitis groups compared to Asp colonisation. Bronchiectasis% clearly was the most important component of SLD, responsible for about 2/3 of all total % disease, while for the other third, a combination of the remaining abnormalities atelectasis%, airway wall thickening%, and mucus plugging% was responsible. The relatively low prevalence of these last three subgroups of SLD make it difficult to evaluate these outcomes separately. Air trapping% was highly prevalent with 57% in the overall study population and no clear difference between the Asp disease phenotypes. Progression per year in air trapping% appeared to be raised only in the disease phenotype of Asp sensitisation, in comparison to the reference group of colonisation. A multivariable model was built to reflect disease progression in the different phenotypes, while adjusting for any PsA and ABPA, and Figure 2 represents the predicted values of this model for the four Asp disease phenotypes for the outcome total % disease.

**Figure 3:**
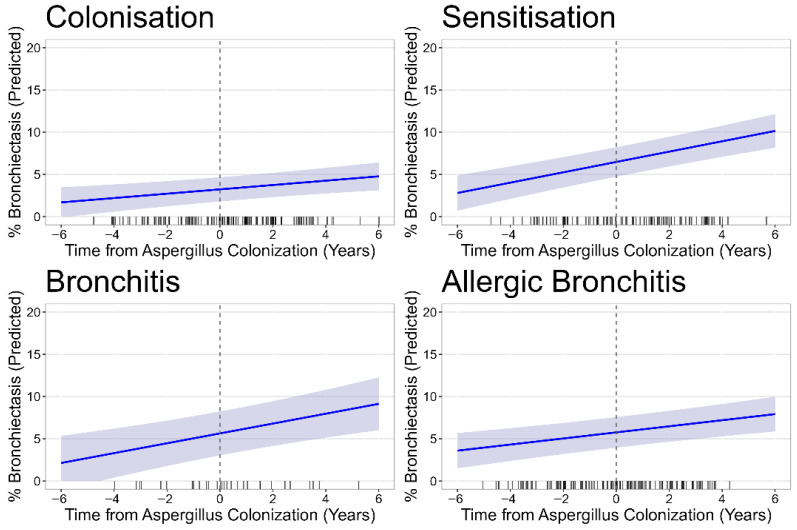
Progression of bronchiectasis% by Asp disease phenotype. The figure displays the modelled trajectory of bronchiectasis% over time, where timepoint 0 = the moment of first Asp+. Each line represents the predicted mean bronchiectasis%, with shaded areas indicating the 95% confidence intervals.

Progression rates remained highly similar to those from the unadjusted stratified analysis reflected in Table 1 for all outcomes and Asp disease phenotypes. The reference group, Asp colonisation, showed a statistically significant progression in total % disease over time, with an average increase of 0.34% per year (CI 0.15, 0.53; *p* < 0.001, Figure A2). In comparison to the Asp colonisation, the rate of total % disease progression was higher in the sensitisation with an additional 0.34% per year (CI 0.06, 0.63, *p* = 0.019) and bronchitis phenotypes (additional 0.49% per year, CI 0.08, 0.90, *p* = 0.021), but the rate in allergic bronchitis was almost identical to colonisation. Exclusion of children who later developed ABPA did not alter the observed progression rates, but statistical significance of the difference between Asp disease phenotypes was lost, possibly due to reduced power. Analysis of the bronchiectasis sub-score yielded very similar results (Figure 3).

**Figure 4:**
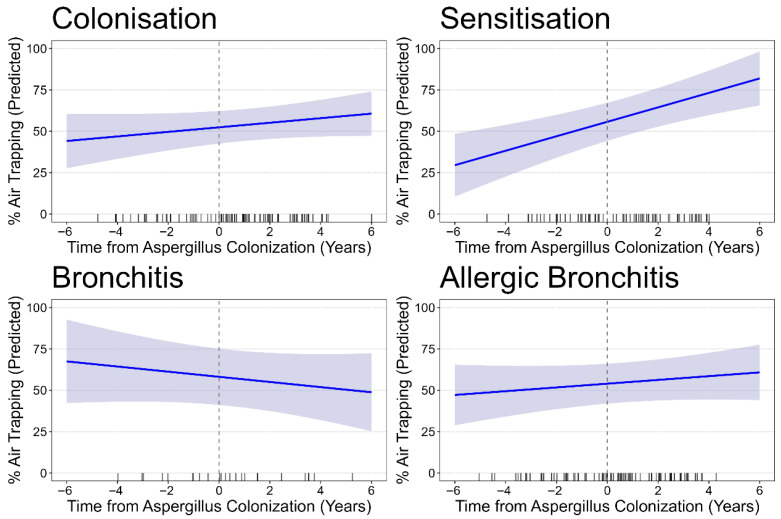
Progression of air trapping% by Asp disease phenotype. The figure displays the modelled trajectory of air trapping% over time, where timepoint 0 = the moment of first Asp+. Each line represents the predicted mean air trapping%, with shaded areas indicating the 95% confidence intervals.

Asp sensitisation, bronchitis, and allergic bronchitis groups all had about double the amount of baseline bronchiectasis scores compared to colonisation. There was progression bronchiectasis% in the Asp colonisation group, with an average annual increase of 0.26% (CI 0.10, 0.42; *p* = 0.002); only Asp sensitisation showed a significantly higher rate of progression over time, with an additional 0.36% per year (CI 0.11, 0.60, *p* = 0.004). Asp bronchitis actually demonstrated a very similar increased rate with 0.33% more progression of bronchiectasis% per year compared to colonisation (CI −0.02, 0.67, *p* = 0.066), but this was not significant likely due to the smaller sample size in this subgroup. No separate adjusted models are presented for the outcomes of atelectasis, airway wall thickening, and mucus plugging, as the low prevalences of these SLD components make it difficult to interpret results by Asp disease phenotype subgroups. Air trapping, assessed only in the subset of patients with available expiratory CTs, was strikingly high across all Asp disease phenotypes (Table 2). Figure 4 shows the predicted values for air trapping over time in the four phenotypes after adjustment for the possible confounders PsA and ABPA.

There was no progression of air trapping over time in the reference group of colonisation. Also, the rate of progression did not differ between the four phenotypes, although Asp sensitisation exhibited a borderline significantly higher annual progression compared to colonisation, with a difference of 3.00% per year (CI 0,00, 5.98, *p* = 0.052).

## 4. Discussion

This is the first study to investigate the impact of Asp infection and its different immunological phenotypes on the progression of SLD in children with CF. Our findings indicate that Asp infection is associated with relatively high prevalence of SLD at baseline and increased rates of progression over time. To our knowledge, this is the first time that the immunological response to Asp, as evidenced by elevated IgG_Asp_ (Asp bronchitis) and/or IgE_Asp_ (Asp sensitisation), can be used to define distinct clinical phenotypes with different levels of baseline SLD and different rates of disease progression over time. Asp sensitisation and bronchitis phenotypes had the highest rate of SLD progression, while the allergic bronchitis phenotype exhibited the highest baseline SLD and was associated with the highest risk of developing ABPA during the study.

Importantly, the rate of disease progression in these children from the Sophia cohort with a positive ASP culture (0.46%/year) was about threefold higher than the average rate of disease in an unselected sample of the same Sophia cohort reported on previously (0.16% per year) [20]. This could be associated with the Asp that was detected, but it may also reflect that Asp is detected more frequently in children with CF with relatively more advanced disease, as reflected by the relatively high proportion of children with any positive PsA culture (77%). We did not find evidence of accelerated SLD progression after the first Asp+ culture was obtained, within our study population. This could imply that Asp positivity is merely a marker of worse lung disease in these children, rather than a causative factor for the progression of structural lung damage, which is an ongoing topic of debate [21,22,23]. It should be noted that the sensitivity of airway cultures in children is considered low, ranging from 14 to 29% for single sputum and bronchoalveolar lavage, up to 54.2% when using high volume of sputum or prolonged culture techniques. Therefore, the first positive culture detected in our study may not be the actual first moment of infection [6,7]. A novel aspect of our study is the separation of different Asp disease phenotypes. Despite small numbers in some subgroups, we found strong evidence for different rates of disease progression according to these phenotypes. This finding supports the notion that Asp can evoke a different immunologic response in different CwCF and that in some of these phenotypes there may indeed be a causal effect of Asp on the progression of SLD. Treatment is generally not considered for the disease phenotype of Asp sensitisation, even though this particular group had higher rates of progression of total disease, bronchiectasis, and air trapping. The differences in SLD progression we report between the different Asp phenotypes cannot easily be explained by confounding factors, such as CF disease severity or presence of PsA, because there is no clear reason why such confounding factors would be associated with differential IgE_Asp_ and IgG_Asp_ responses.

One of the most severe pulmonary immunological responses to Asp is ABPA, which can have severe clinical symptoms and radiological sequalae, and for which there is clear consensus that treatment is indicated [24,25]. There is evidence from case series for the use of steroids [14], and recently the pragmatic use of anti-fungal has become quite common. Case series have reported beneficial effects of anti-fungal, even though there are adverse effects associated with their long-term use [12]. Our finding that in comparison to colonisation, the phenotypes of sensitisation and bronchitis had a fourfold higher risk, and allergic bronchitis even a more than tenfold increased risk of developing ABPA may be of clinic relevance. More frequent follow-up, earlier identification, and possibly even preventative anti-fungal treatment could all be considered in those at highest risk of developing ABPA. We found it surprising that the group of allergic bronchitis, with both an allergic and immune response to Asp did not experience the highest rate of SLD progression over time. We hypothesise this association may, to some extent, be confounded by treatment effects. During the study period at our clinical centre, ABPA was the only generally accepted indication for aggressive treatment of both Asp directly with anti-fungal and ABPA with high-dose steroids. Hence, the relatively high percentage of ABPA (31%) that occurred well within the study period (median 7.5 months after Asp+) may have offered some protection in this group for the long-term sequelae of Asp on SLD.

Our study has some clear limitations that need to be considered. It was a retrospective study, which means that we could only use clinically collected and stored data of the routine annual visits. Missing data may have introduced some selection bias, further limiting generalisability of this single-centre study. To improve the sensitivity of Asp detection we accepted all fungal airway cultures, while others chose to only investigate positive bronchoalveolar lavage samples of the lower airways [10]. The timing of blood samples used to define Asp disease phenotypes was limited to annual visits. The small sample size, particularly in the Asp bronchitis subgroup, reduces statistical power to detect differences in progression rates and limits the number of confounders that could be considered. Furthermore, the limited sample size limited the ability to correct for a wide range of confounders. We did include PsA as it has been noted that treatment of PsA may be associated with Asp culture positivity and that disease progression in these CwCF may in fact be caused by PsA rather than Asp [11]. However, only the rather crude measure of ‘any positive PsA culture during the study’ could be collected and corrected for. Adjustment of our models for PsA had little to no effect on the reported associations between Asp and SLD in any of the Asp disease phenotypes. Finally, in our analysis we specifically and exclusively investigated *Aspergillus fumigatus*, as this is considered the most prevalent and relevant fungus in CF lung disease. We do not have any information on other fungal pathogens in our study population. We propose that despite these clear limitations, our study provides some novel insights and support for the existence of different Asp disease entities with clear clinical relevance.

There is ongoing debate about the indications to treat Asp lung disease due to the uncertainty of clinical benefit, difficult and prolonged treatment regimens, as well as the relatively high toxicity and risk for side effects [26,27]. The choice to initiate anti-fungal treatment remains a difficult and case-by-case decision, based on expected benefits and risks. In this trade-off, the recent widespread introduction of triple combination modulator therapies needs to be considered [28,29,30]. We assume our study was minimally affected by modulator use as only 5% of CTs were performed on double combination and none on the much more effective triple combination therapy. We can only hypothesise how the historic rates of SLD progression in Asp phenotypes will translate to the post-modulator era. There remains a group of CwCF who are not eligible or responsive to modulator therapy and even CwCF on modulators and their clinicians will be confronted with positive cultures and the question of how to deal with this. Based on our results, we propose that a thorough evaluation of the Asp phenotype may give some guidance in this difficult decision, in order to take the potentially higher risk for SLD progression or the development of ABPA into account.

## 5. Conclusions

This study demonstrates that the immune-based Asp phenotypes of colonisation, sensitisation, bronchitis, and allergic bronchitis each convey distinct risks for SLD progression in CwCF. These findings offer support for the hypothesis that some Asp phenotypes are causally related to the accelerated progression of SLD in CwCF. Assessing Asp phenotype at the moment an airway culture becomes positive for the first time may help to assess risks of further sequalae and guide difficult treatment decisions.

## Figures and Tables

**Table 1:** Patients descriptives in overall population and by Asp disease phenotype.

Descriptive	All Patients	Asp Colonisation	Asp Sensitisation	Asp Bronchitis	Asp Allergic Bronchitis
		IgE_Asp_ − IgG_Asp_ −	IgE_Asp_ + IgG_Asp_ −	IgE_Asp_ − IgG_Asp_ +	IgE_Asp_ + IgG_Asp_ +
Number of patients	125	44	32	10	39
Number of scans	382	131	102	30	119
Male gender (n, %)	59 (47.2%)	20 (45.4%)	14 (31.8%)	5 (50%)	20 (52.3%)
Age first Asp+ culture (yrs) (median [IQR])	9 [6]	6.5 [8.25]	10 [5]	8.5 [4.5]	11 [4]
Age first included CT (yrs) (years: median [IQR])	7 [5]	6 [7]	7.5 [5]	4.5 [6]	8 [3]
Age last included CT (yrs) (median [IQR])	12 [7]	9 [7.25]	13.5 [5.25]	11 [2.5]	13 [3.8]
Any positive PsA culture (n, %)	96 (76.8%)	28 (63.6%)	26 (81.3%)	9 (90.0%)	33 (84.6%)
Developed ABPA (n, %) *	17 (13.6%)	1 (2.3%)	3 (9.4%)	1 (10.0%)	12 (30.7%)
Time between first Asp+ and diagnosis ABPA (months) (median [IQR])	10 [21]	60	5 [22.5]	19	7.5 [16]

Plus sign implies levels raised above upper limit of normal, minus sign implies within normal range. Asp colonisation (both IgE_Asp_ and IgG_Asp_ normal), Asp sensitisation (IgE_Asp_ elevated, IgG_Asp_ normal), Asp bronchitis (IgE_Asp_ normal, IgG_Asp_ elevated), Asp allergic bronchitis (both IgE_Asp_ and IgG_Asp_ elevated), PsA = *Pseudomonas aeruginosa*, ABPA = Allergic Bronchopulmonary Aspergillosis. IQR = Interquartile range. *: *p*-value for Anova (continuous variables) or Chi-square test (dichotomous variables) < 0.05.

**Table 2:** Prevalence at the time of first ASP+ and annual progression rates of SLD components by Asp disease phenotype.

		Total % Disease	Bronchiectasis%	Atelectasis%	Airway Wall Thickening%	Mucus Plugging%	Air Trapping%
Full study population		125 pts with 382 CTs					105 pts; 266 CTs
	At first Asp+	8.51 (7.59, 9.43)	6.16 (5.45, 6.86)	0.88 (0.70, 1.07)	1.04 (0.97, 1.12)	1.27 (0.98, 1.56)	57.49 (53.14, 61.84)
	Progression/year	0.46 (0.35, 0.57)	0.42 (0.32, 0.51)	−0.05 (−0.11, 0.01)	0.01(−0.01, 0.03)	0.04 (−0.02, 0.10)	1.53 (0.40, 2.67)
Asp colonisation		44 pts; 131 CTs					37 pts; 90 CTs
	Baseline	5.92 (5.09, 6.75)	4.35 (3.66, 5.04)	0.81 (0.50, 1.11)	0.99 (0.87, 1.11)	0.58 (0.36, 0.81)	53.60 (44.59, 62.61)
	Progression/year	0.31 (0.13, 0.49)	0.24 (0.08, 0.39)	−0.08 (−0.2, 0.05)	0.04 (0.001, 0.08)	0.02 (−0.04, 0.08)	1.53 (−0.42, 3.48)
Asp sensitisation		32 pts; 102 CTs					25 pts; 67 CTs
	Baseline	10.16 (8.61, 11.70)	7.73 (6.48, 8.98)	0.65 (0.44, 0.85)	1.00 (0.85, 1.15)	1.34 (0.82, 1.86)	57.65 (51.10, 64.20)
	Progression/year	0.70 (0.47, 0.92)	0.62 (0.43, 0.80)	0.02 (−0.01, 0.05)	−0.01 (−0.05, 0.02)	0.12 (−0.01, 0.24)	4.30 (1.75, 6.85)
Asp bronchitis		10 pts; 30 CTs					8 pts; 25 CTs
	Baseline	10.15 (0.80, 13.49)	7.07 (4.62, 9.51)	0.87 (−0.06, 1.80)	1.29 (1.03, 1.55)	1.72 (0.52, 2.92)	62.01 (53.01, 71.02)
	Progression/year	0.85 (0.57, 1.13)	0.60 (0.34, 0.86)	0.11 (−0.04, 0.25)	−0.03 (−0.11, 0.05)	0.23 (0.01, 0.46)	−2.38 (−5.59, 0.082)
Asp allergic bronchitis		39 pts; 119 CTs					35 pts; 84 CTs
	Baseline	9.59 (7.48, 11.70)	6.65 (5.09, 8.22)	1.14 (0.74, 1.55)	1.08 (0.09, 1.23)	1.81 (1.12, 2.50)	57.85 (50.37, 65.33)
	Progression/year	0.28 (0.07, 0.50)	0.36 (0.18, 0.54)	−0.06 (−0.18, 0.07)	0.01 (−0.03, 0.04)	−0.06 (−0.20, 0.90)	1.29 (−0.33, 2.92)

Values represent means (95% CI) from unadjusted mixed-effect models in the overall study population and in the stratified groups of the different Asp disease phenotypes. pts = Patients.

## Data Availability

The original contributions presented in this study are included in the article. Further inquiries can be directed to the corresponding author.

## Abbreviations

ABPA	Allergic Bronchopulmonary Aspergillosis
Asp	*Aspergillus fumigatus*
Asp+	Positive *Aspergillus fumigatus* Culture
AT	Air Trapping
CF	Cystic Fibrosis
CT	Computed Tomography
CwCF	Children with Cystic Fibrosis
IgE_Asp_	Specific *Aspergillus fumigatus* IgE
IgG_Asp_	Specific *Aspergillus fumigatus* IgG
PRAGMA	Perth Rotterdam Annotation Grid Morphometric Analysis
PsA	*Pseudomonas aeruginosa*
Pts	Patients
SLD	Structural Lung Disease
